# Excellent Thermoelectric Performance of 2D CuMN_2_ (M = Sb, Bi; N = S, Se) at Room Temperature

**DOI:** 10.3390/ma15196700

**Published:** 2022-09-27

**Authors:** Wenyu Fang, Yue Chen, Kuan Kuang, Mingkai Li

**Affiliations:** 1 Ministry-of-Education Key Laboratory of Green Preparation and Application for Functional Materials, Hubei Key Lab of Ferro & Piezoelectric Materials and Devices, Hubei Key Laboratory of Polymer Materials, and School of Materials Science & Engineering, Hubei University, Wuhan 430062, China; 2 Public Health and Management School, Hubei University of Medicine, Shiyan 442000, China

**Keywords:** 2D material, conductivity, power factor, spin-orbit effects, figure of merit

## Abstract

2D copper-based semiconductors generally possess low lattice thermal conductivity due to their strong anharmonic scattering and quantum confinement effect, making them promising candidate materials in the field of high-performance thermoelectric devices. In this work, we proposed four 2D copper-based materials, namely CuSbS_2_, CuSbSe_2_, CuBiS_2_, and CuBiSe_2_. Based on the framework of density functional theory and Boltzmann transport equation, we revealed that the monolayers possess high stability and narrow band gaps of 0.57~1.10 eV. Moreover, the high carrier mobilities (10^2^~10^3^ cm^2^·V^−1^·s^−1^) of these monolayers lead to high conductivities (10^6^~10^7^ Ω^−1^·m^−1^) and high-power factors (18.04~47.34 mW/mK^2^). Besides, as the strong phonon-phonon anharmonic scattering, the monolayers also show ultra-low lattice thermal conductivities of 0.23~3.30 W/mK at 300 K. As results show, all the monolayers for both p-type and n-type simultaneously show high thermoelectric figure of merit (*ZT*) of about 0.91~1.53 at room temperature.

## 1. Introduction

Thermoelectric generators can directly convert heat into electrical power, thus attracting wide research interest. Generally, the thermal-electric conversion capacity can be ruled by the dimensionless figure of merit, $ZT=\frac{\sigma S^{2}T}{\kappa_{e}+\kappa_{l}}$ [1], here *S* and σ are the Seebeck coefficient and electrical conductivity, *T* presents the temperature, $\kappa_{e}$ and $\kappa_{l}$ are the electron and lattice thermal conductivity, respectively. Clearly, the ideal thermoelectric material needs to have both high-power factor ($PF=\sigma S^{2}$) and low lattice thermal conductivity. However, this target is not easy to achieve simultaneously as the parameters above are tightly coupled, mutually restricted, and difficult to decouple. They can be regarded as the functions of the vector tensor ${Κ}_{n}$, energy eigenvalue $\varepsilon_{i}$, and carrier relaxation time $\tau_{i}\left(k\right)$ [2,3,4]. Besides, the Seebeck coefficient is also closely related to the density of states effective mass ($m_{d}^{*}$) and intrinsic carrier concentration (*n*), $S=\frac{8\pi^{2}k_{B}^{2}Tm_{d}^{*}}{3e\hbar^{2}}{\left(\frac{\pi }{3n}\right)}^{2/3}$ [5]. Additionally, electrical conductivity σ, and electron thermal conductivity $\kappa_{e}$ are also restricted by the Wiedemann-Franz-Lorenz’s law, $\kappa_{e}=L\sigma T$ [6], where *L* is Lorenz number.

In fact, the thermoelectric performance of traditional thermoelectric materials has been effectively improved over the past few decades. Among them, two-dimensional layered (2D) materials, as unique mechanical, electronic, thermal, and optoelectronic properties, as well as quantum confinement effects, make them as promising thermoelectric materials in a variety of applications. For example, quasi-two-dimensional SnSe transistors were revealed to have high Seebeck coefficient, and a field effect mobility of about 250 cm^2^/Vs at 1.3 K, thus it was found to be a high-quality semiconductor ideal for thermoelectric applications [7]. The 2D Mg_3_Sb_2_ monolayer was proved to have a favorable *ZT* value of 2.5 at 900 K, which is higher than that of its bulk structure. These theoretical results also revealed that nano-engineering can effectively improve the thermoelectric conversion efficiency [8]. Additionally, quasi-two-dimensional GeSbTe compounds were observed by Wei et al. [9]. They found that the monolayer with maximal *ZT* values of 0.46~0.60 at 750 K, indicating that 2D GeSbTe is a promising mid-temperature thermoelectric material.

Recently, research on the thermoelectric properties of copper-based semiconductors has attracted much attention. For example, Yu et al. [10] proposed a novel phrase 2D *σ*-Cu_2_S, which has a low lattice thermal conductivity of 0.10 W/mK, and a high *ZT* value of 1.33 at 800 K. Cao et al. [11] investigated the electronic structure and thermoelectric performance of *β*-Cu_2_Se under strain of −4~4%, they found that its *ZT* values can reach 1.65~1.71 at 800 K. Other materials, such as Fm-3m Cu_2_S [12], multi-scale Cu_2_Se [13], and Cu-Se co-doped Ag_2_S [14], all exhibited intrinsic low thermal conductivity and high *ZT* value. In fact, back in 2013 and 2016, Ma and Deng et al. [15,16] investigated the diffusion behavior of Cu in CdTe by density functional theory, they found that Cu s-d orbital coupling only occurred at asymmetric point, but instantly disappeared at symmetric location. These interesting properties make Cu based compound to possess a strong anharmonic scattering, resulting in low intrinsic lattice thermal conductivity. In addition, many semiconductors containing metallic atoms such as Bi have also been reported to possess non-negligible spin-orbit coupling (SOC) effects [17,18], which have the potential to be used in thermoelectric devices. For example, Kim et al. [19] prepared the bulk Bi_0.4_Sb_1.6_Te_3_ alloy via an atomic-layer deposition (ALD) technique, and found that it possesses high *ZT* values of 1.50 at 329 K. Wu et al. [20] investigated the thermoelectric properties of *β*-BiAs and *β*-BiSb monolayers by first-principles calculation and Boltzmann transport theory. They concluded that the monolayers simultaneously exhibit ultra-low lattice thermal conductivities (0.6~0.8 W/mK), and high *ZT* values (0.78~0.82) at 300 K.

CuMN_2_ (M = Sb, Bi; N = S, Se) are layered materials with narrow band gaps within ~1.38 eV. [21] Among them, CuSbS_2_ exhibited the low lattice thermal conductivity of about 1.5~2 W/mK [22]. Additionally, both bulk and monolayer CuSbS_2_ and CuSbSe_2_ were revealed to have an excellent thermoelectric power factor at 300 K, reaching about 0.2~1.0 mW/mK^2^ at constant relaxation time approximation (CRTA) of 11 fs [23]. Therefore, it is of high interest to see whether or not the monolayer CuSbS_2_, CuSbSe_2_, CuBiS_2_, and CuBiSe_2_, can also deliver good thermoelectric performance. To this end, using first principles calculations, we investigated the electronic structures, mechanical, and transport properties, of these four 2D materials. The calculations revealed that all the monolayers possess narrow band-gaps (0.57~1.10 eV), high power factors (18.04~47.34 mW/mK^2^), and also low lattice thermal conductivities (0.23~3.30 W/mK). As a result, all the monolayers for both p-type and n-type simultaneously show high *ZT* values of about 0.91~1.53 at room temperature.

## 2. Calculation Details

We carried out the calculations in the Vienna Ab initio Simulation Package (VASP) [24], in which the Generalized Gradient Approximation (GGA) [25,26] and Perdew–Burke–Ernzerhof (PBE) functional was adopted to exchange-correlation approximation. To shorten the computation time, we used the HipHive [27] code to extract the second and third order force-constants (IFCs). Besides, we used the Phonopy [28] code to calculate the Grüneisen parameters and phonon dispersion, and employed the Phono3py [29] to evaluate the phonon scattering rate and lattice thermal conductivity. We also used the Wannier90 [30] code to solve Boltzmann transport equation, and then characterized the thermoelectric properties as a function of the chemical potential.

The transport properties, such as electrical conductivity, electron thermal conductivity and *ZT* value, are directly related to carrier relaxation time, therefore, we used the deformation potential theory (DPT) to calculate carrier mobility, and further corrected it by the acoustic phonon-limited method (APM), which is more suitable for anisotropic materials. See Supplementary Materials for more calculation details.

## 3. Results and Discussion

### 3.1. Crystal Structures

**Figure 1:**
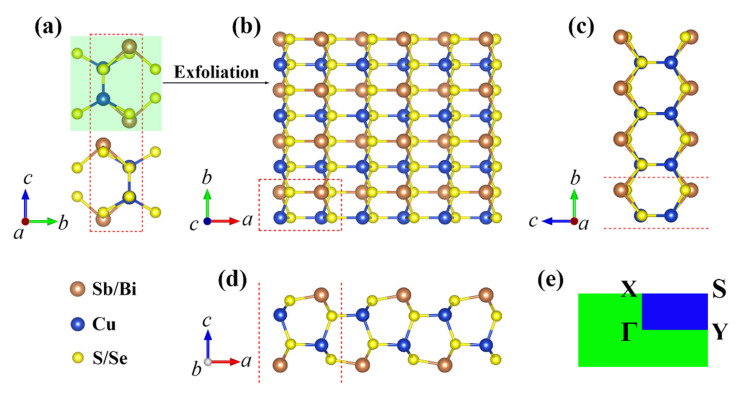
The structures of: (**a**) bulk; (**b**) top view; (**c**,**d**) side view; and (**e**) K-point path of 2D CuMN_2_ (M = Sb, Bi; N = S, Se).

The crystal structures of CuMN_2_ (M = Sb, Bi; N = S, Se) for bulk and single-layer are shown in Figure 1. As can be seen, the bulk CuMN_2_ are layered structures, similar to graphite and MoS_2_. Therefore, we first investigated the cleavage energy of their single-layers, and its calculation method was based on the latest Rigorous Method proposed by Jung et al. [31]. $E_{f}=\frac{E_{\mathrm{iso}}-E_{\mathrm{bulk}}/n}{A}$, where $E_{\mathrm{iso}}$ and $E_{\mathrm{bulk}}$ are energies of single-layer and bulk unit-cell, A and n are the in-plane area and the number of the slab in a bulk-unit. The calculation results are listed in Table S1. The corresponding cleavage energies are within 0.68~0.93 J/m^2^, which are higher than those of graphene (0.33 J/m^2^), black phosphorus (BP) (0.36 J/m^2^), and MoS_2_ (0.27 J/m^2^) [32], but still lower than those of single-layer Ca_2_N (1.09 J/m^2^) [33], GeP_3_ (1.14 J/m^2^), and InP_3_ (1.32 J/m^2^) [34]. All of these results indicate the feasibility of obtaining single-layer CuMN_2_ by mechanical exfoliation in experiments. The lattice constants and thicknesses of the monolayers after structural relaxation are listed in Table 1. Owing to each atomic radius satisfies: S (1.03 Å) < Se (1.16 Å) < Cu (1.28 Å) < Sb (1.61 Å) < Bi (1.82 Å), both the lattice constants (*a*/*b*) and thickness (*h*) follow the order of CuSbS_2_ < CuBiS_2_ < CuSbSe_2_ < CuBiSe_2_.

### 3.2. Elastic Properties and Stability

**Figure 2:**
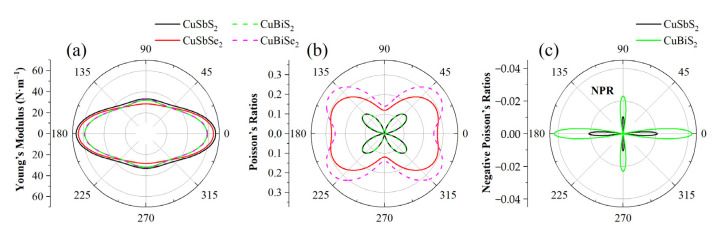
(**a**) The Young’s moduli: (**b**) Poisson’s ratio; and (**c**) NPR of 2D CuMN_2_ (M = Sb, Bi; N = S, Se).

In general, for a new 2D material, we can identify its mechanical stability by its elastic constants $C_{ij}$. As listed in Table 2, all the monolayers satisfy the Born-Huang criterion, $C_{11}C_{22}-C_{12}^{2}>0$ and $C_{66}>0$ [35], indicating that they all possess high mechanical stability. Additionally, since the structures of the monolayers are anisotropic, $C_{11}\neq C_{22}$. We further calculated the Young’s moduli and Poisson’s ratio of these materials [36], as shown in Figure 2. Here θ is the angle with respect to *a*-axis. As can be seen, their Young’s moduli are relatively close, showing the maximum values of 58.20~66.52 N/m in the direction of 0° (180°) and the minimum values of 28.34~33.04 N/m in the direction of 90° (270°), which are obviously lower than those of graphene (350 ± 3.15 N/m) [37], h-BN ((270 N/m), [38] and MoS_2_ (200 N/m) [39]. Such low Young’s moduli are expected to exhibit low lattice thermal conductivity [40]. On the contrary, the Poisson’s ratio minimizes in both 0° and 90° directions, and maximizes at 40° (140°) with values of 0.14~0.34, respectively. Fantastically, monolayer CuSbS_2_ and CuBiS_2_ are rare auxetic materials with negative Poisson’s ratio (NPR) of −0.02 and −0.04 at 0° (180°). Such interesting NPR phenomenon is also observed in PN (−0.08) [41], and tetra-silicene (−0.06) [42], which have been revealed to hold high potential in medicine, defense, and the escalation of tensions [43].

We also investigated the bonding strength of materials by calculating their electron localization function (ELF) and Bader charge analysis (see Figure S1 and Table S2 for more details). Clearly, the adjacent atoms exhibit a predominant ionic bond characteristic. However, the net charge transfer is relatively few, mostly within ~1.0 *e*, indicating that they have relatively weak bond strength and thus exhibit low Young’s moduli. Besides, we analyzed the thermal stabilities of these four materials at different temperatures by using ab initio molecular dynamics (AIMD) simulations [44]. We revealed that they can remain high stability at 500 K, as their crystal structure does not bond breaking or undergo remodeling, as showed in Figure S2 in Supplementary Materials.

### 3.3. Electronic Structures

**Figure 3:**
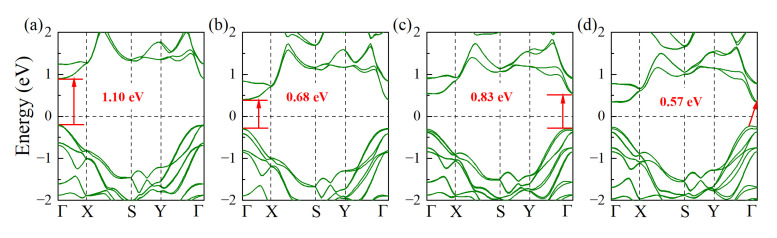
Band structures of monolayer: (**a**) CuSbS_2_; (**b**) CuSbSe_2_; (**c**) CuBiS_2_; and (**d**) CuBiSe_2_ at HSE 06 + SOC functional.

**Figure 4:**
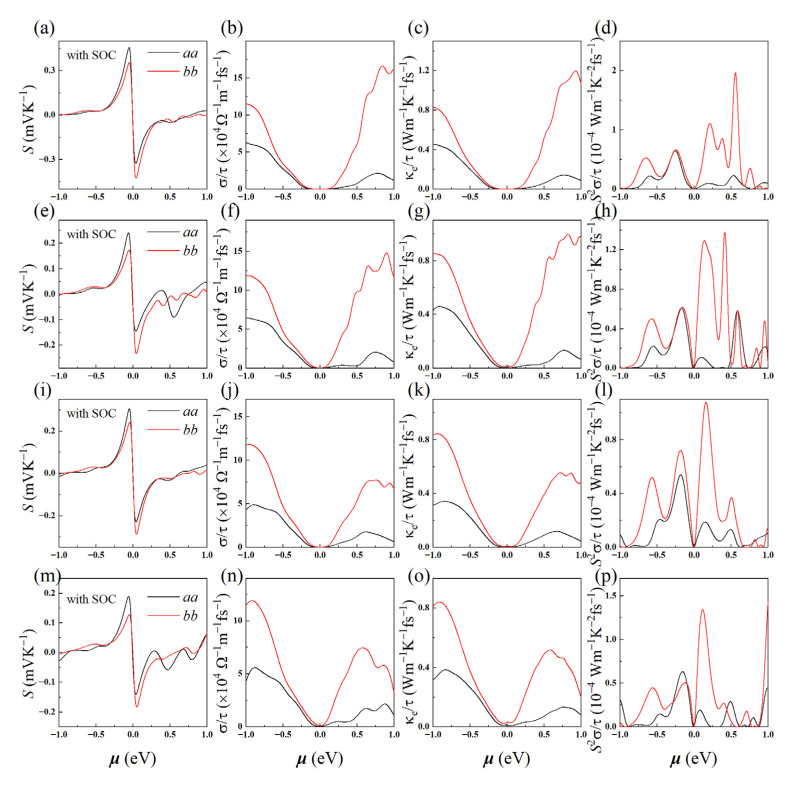
Electron transport properties of 2D CuMN_2_ (M = Sb, Bi; N = S, Se): (**a**,**e**,**i**,**m**) Seebeck coefficients; (**b**,**f**,**j**,**n**) electrical conductivities; (**c**,**g**,**k**,**o**) electron thermal conductivities; and (**d**,**h**,**l**,**p**) power factors along *a*—(black line) and *b*—directions (red line) with PBE + SOC functional.

To accurately characterize the electronic structures of the monolayers, we first analyze the effect of spin-orbit coupling (SOC) on their electronic band structures. As shown in Figure S3, SOC has a non-ignorable impact on band structure, especially for CuBiS_2_ and CuBiSe_2_, so SOC was all considered in following calculations. As shown in Figure 3, all the monolayers are narrow band-gap semiconductors with band gaps of 0.57~1.10 eV, which are slightly smaller than or comparable to their bulk structures [21,23,45,46]. Since the valence band maximum (VBM) and conduction band minimum (CBM) are both located at Γ point, both CuSbS_2_, CuSbSe_2_, and CuBiS_2_ belong to direct bandgap semiconductors. However, for CuBiSe_2_, its VBM is transferred to between Y and Γ, so it is an indirect bandgap semiconductor. Moreover, as show in Figure S4, the VBMs are mainly composed of S-3p/Se-4p and Cu-3d electrons, while CBMs are mainly composed of Sb-5p/Bi-6p and S-3p/Se-4p electrons. In addition, the total density of states for both VBMs and CBMs showed relatively steep distribution, indicating that the monolayers have high density of states effective mass, and thus to possess both high p-type and n-type Seebeck coefficients, as shown in Figure 4.

Next, we explored the carrier mobility of electrons and holes along the *a*- and *b*-axis of the monolayers (see Figures S5 and S6, and Table S3 for more details). For monolayer CuSbS_2_, CuSbSe_2_, and CuBiS_2_, their effective masses (*m_e_*) of electrons are higher than those of holes (*m_h_*) along *a*-axis, corresponding to their flatter band near CBMs than VBMs along the Γ–X direction. However, the opposite is true for CuBiSe_2_, the *m_e_* (0.93 *m*_0_) is indeed lower than *m_h_* (2.83 *m*_0_), which can be interpreted as the steeper dispersion curve corresponding to its VBM between Γ and X. Besides, for all monolayers, their elastic modulus (*C*^2D^) along the *a*-axis is larger than that along *b*-axis, which is consistent with their elastic constants. Further, the deformation potential constant (*E_l_*) fluctuates widely from 0.75 eV to 4.48 eV, and the lower *E_l_* occur simultaneously for the hole along the *a*-axis. As a result, the hole mobilities in the *a*-axis are higher than those in other cases, and the highest is even up to 4562.47 cm^2^·V^−1^·s^−1^ for CuSbS_2_. Since DPT method tends to overestimate the mobility of semiconductor, especially when the *E_l_* is relatively small [35], we adopted the APT method to correct the results, as listed in Table 3. Clearly, after corrected by APT, the carrier mobility increases when the *E_l_* is relatively large, and decreases otherwise. As can be seen, the mobilities of both electrons (*μ_e_*) and holes (*μ_h_*) are basically in the range of 10^2^~10^3^ cm^2^·V^−1^·s^−1^, in which CuSbS_2_ and CuSbSe_2_ exhibit the highest *μ_h_* and *μ_e_* of 1661.49 and 937.12 cm^2^·V^−1^·s^−1^, which are far higher than that of MoS_2_ (*μ*_h_ ~200 cm^2^·V^−1^·s^−1^) [47], but lower than those of silicene (*μ*_e_ ~10^5^ cm^2^·V^−1^·s^−1^) [48], and phosphorene (*μ*_h_ ~10^4^ cm^2^·V^−1^·s^−1^) [49].

### 3.4. Electrical Transport Properties

Furthermore, we explored the electron transport properties of the monolayers by solving Boltzmann transport equation, as show in Figure 4 (see Figure S7 for more details about maximally localized Wannier functions (MLWFs)). Obviously, the maximums of Seebeck coefficient (*S*) satisfy CuSbS_2_ > CuBiS_2_ > CuSbSe_2_ > CuBiSe_2_, which consist with their band-gaps ordering, as small gap implies high carrier concentration. Coincidentally, the p-type Seebeck coefficients are higher than those of n-type in *a*-axis, but opposite in the *b*-axis, which may be caused by the anisotropy of the density of states effective mass. Besides, constrained by the Wiedemann-Franz-Lorenz’s law [3], the electronic thermal conductivity and electrical conductivity have similar curves. In general, electron transport properties are directly related to the carrier relaxation time (τ). Therefore, we further calculated it by using the formula $\tau =\frac{m^{*}\mu }{e}$ [50], here $m^{*}$, μ, and *e* are carrier effective mass, mobility, and electron charge, as listed in Table 3. After taking the relaxation time, the monolayers exhibit high electrical conductivity of up to 10^6^~10^7^ Ω^−1^·m^−1^, also high *PF* of 18.04~47.34 mW·K^−2^·m^−1^ at 300 K (see Table 4), which are higher than or comparable to those of their bulk structures (0.8~1.0 mW/mK^2^) [23], Pd_2_Se_3_ (1.21~1.61 mW·K^−2^·m^−1^) [6], PdSe_2_ (5~25 mW·K^−2^·m^−1^) [51], and Tl_2_O (10~33 mW·K^−2^·m^−1^) [52]. 

### 3.5. Phonon Transport Properties

**Figure 5:**
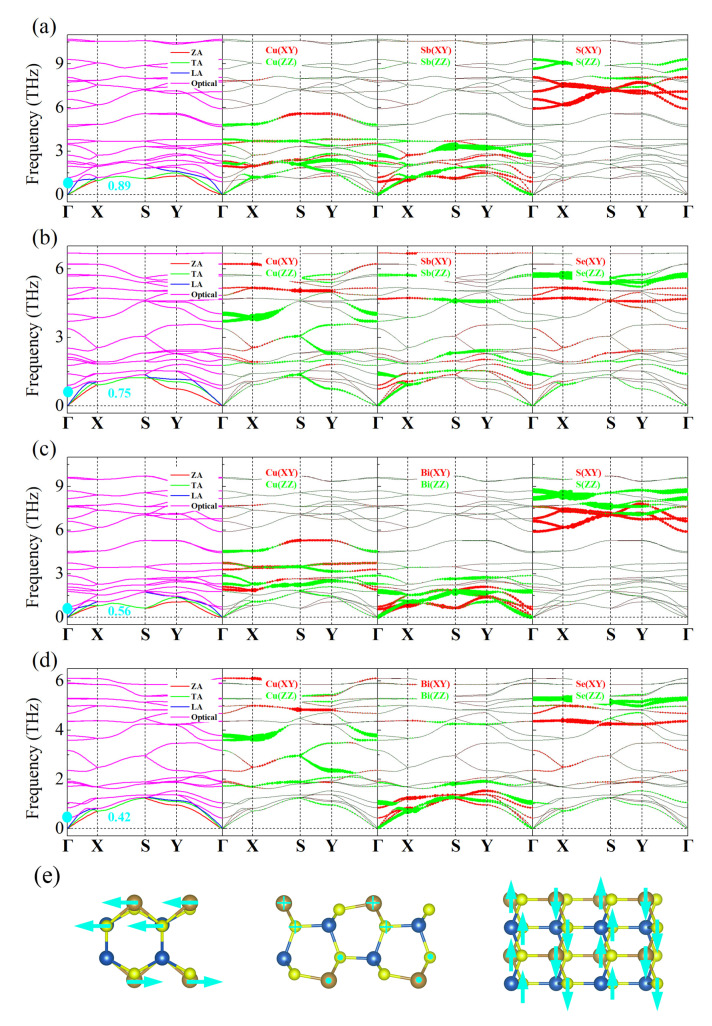
The orbital-resolved phonon dispersion of: (**a**) CuSbS_2_; (**b**) CuSbSe_2_; (**c**) CuBiS_2_; (**d**) CuBiSe_2_; where the XY and ZZ denote the in-plane and out-of-plane phonons; and (**e**) the schematic diagram of low frequency optical phonon at Γ point for monolayer CuSbS_2_, where the arrows represent the direction of the vibration.

The phonon dispersions are shown in Figure 5, where the red, green, blue, and pink curves denote the out-of-plane acoustic (ZA), longitudinal acoustic (LA), transverse acoustic (TA), and optical phonons, respectively. As can be seen, these phonon dispersions have no virtual frequencies, indicating that these four monolayers have high kinetic stability. As the Sb(Bi) atoms are heavier than the others, they show lower phonon frequencies, while the lighter S(Se) atoms possess higher frequencies. Meanwhile, there is some coupling between in-plane (XY) and out-of-plane phonons (ZZ) for all atoms, which can be attributed to the fact that each atom is dispersed in multiple layers (see Figure 1), which breaks the plane symmetry of their structure and allows more phonons to participate in scattering [53]. Additionally, the lowest optical mode boundary frequencies at Γ point of the monolayers are within 0.42~0.89 THz, which are close to those of SnSe (~0.99 THz) [54], KAgS (~1.20 THz) [50], and PbSe (~0.63 THz) [55], indicating that their optical modes softening is relatively severe, as in these materials with intrinsic low thermal conductivity. Further, the low-frequency optical modes at Γ points are caused by the antiparallel motions of the outer Sb/Bi and S/Se atoms, which can effectively increase the phonon dissipation and further reduce the phonon lifetime [55]. Comparatively, for monolayer CuSbSe_2_ and CuBiSe_2_, their phonon frequencies are relatively lower, and more coupling occurs in the low frequency range, resulting in their scattering free path is shorter, and thus have lower phonon lifetimes.

**Figure 6:**
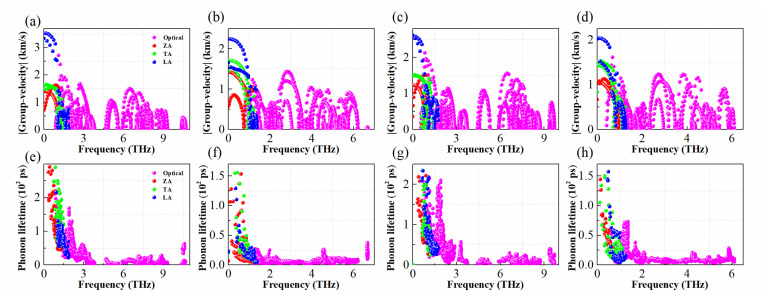
Group velocities (**a**–**d**) and phonon lifetimes (**e**–**h**) of 2D CuMN_2_ (M = Sb, Bi; N = S, Se), here the red, blue, green, and pink dot correspond to ZA, LA, TA, and Optical phonons, respectively.

The lattice thermal conductivity, $k_{l}=\sum_{\lambda }c_{ph,\lambda }{\nu_{\alpha ,\lambda }}^{2}\tau_{\lambda }$, can be expressed as the volumetric specific heat $c_{ph,\lambda }$, group velocity $\nu_{\alpha ,\lambda }$, and phonon lifetime $\tau_{\lambda }$, respectively. The group velocity $\nu_{\alpha ,\lambda }=\partial \omega \left(q\right)/\partial q$, can also be calculated by the first derivative of frequency $\omega \left(q\right)$ with respect to the wave vector *q* [38]. As seen in Figure 6a–d, the LA modes for all the monolayers exhibit maximum group velocities of 2.06~3.59 km/s, smaller than those of Arsenene and Antimonene (~4.5 km/s) [56], BP (~8.6 km/s) [57], and MoS_2_ (~6.5 km/s) [58]. Although the optical modes also exhibit large group velocities in high frequency region, their phonon lifetime is very small, almost zero, as shown in Figure 6e–h, so the $k_{l}$ of these monolayers are mainly contributed by acoustic modes. In addition, for monolayer CuSbSe_2_ and CuBiSe_2_, their phonon lifetimes are significantly shorter than those of the others, which is mainly due to their strong coupling at low frequency phonons, as analyzed above.

**Figure 7:**
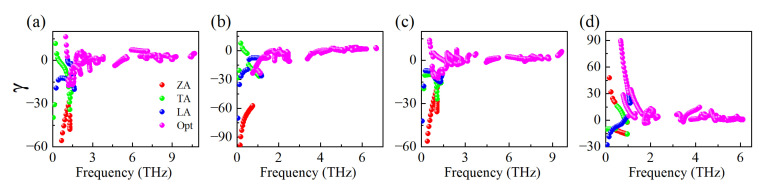
The Grüneisen parameters γ for monolayer: (**a**) CuSbS_2_; (**b**) CuSbSe_2_; (**c**) CuBiS_2_; and (**d**) CuBiSe_2_.

Generally, we can use the Grüneisen parameters γ to describe the anharmonic interactions of a material, which is effective mean to analyze the physical nature of lattice thermal conductivity. It can be obtained from the relationship of phonon frequency $\omega \left(q\right)$ and volume V as $\gamma =\frac{V}{\omega \left(q\right)}\frac{\partial \omega \left(q\right)}{\partial V}$ [59]. For a large $\left|\gamma \right|$ indicates the strong phonon-phonon anharmonic scattering, resulting in a low intrinsic $k_{l}$. As shown in Figure 7, all the monolayers exhibited the high $\left|\gamma \right|$ in the low frequency range, which are similar to that of KAgX (X = S, Se) [50]. Obviously, CuSbSe_2_ and CuBiSe_2_ exhibited larger values than those of CuSbS_2_ and CuBiS_2_, and thus have inherently stronger anharmonic interactions, as well as lower $k_{l}$. Moreover, the negative γ indicate that these materials may have negative thermal expansion (NTE) properties [4]. 

**Figure 8:**
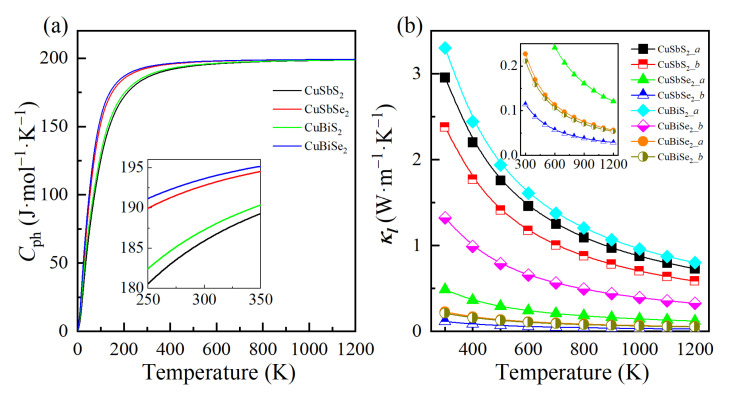
(**a**) The volumetric specific heat, and (**b**) lattice thermal conductivity of 2D CuMN_2_ (M = Sb, Bi; N = S, Se).

Although the volumetric specific heat $c_{ph,\lambda }$ is also directly related to the $k_{l}$, the difference is very small, especially as the temperature increases, as seen in Figure 8a. As a result, all the materials exhibit low $k_{l}$ within ~3.30 W·m^−1^·K^−1^, with CuSbSe_2_ and CuBiSe_2_ having lower values due to stronger phonon anharmonic interactions and low phonon lifetimes. Additionally, we can notice that the $k_{l}$ is higher in *a*-axis, which can be attributed to the stronger bonding, as well as higher Young’s moduli in this direction, and thus better heat transport. As shown in Figure 8b and Table 4, the monolayers show the low lattice thermal conductivities of 0.23~3.30 W·m^−1^·K^−1^ at 300 K, which are comparable to or lower than those of bilayer SnSe (0.9 W·m^−1^·K^−1^) [60], Tl_2_O (0.9~1.2 W·m^−1^·K^−1^) [52], Tetradymites (1.2~2.1 W·m^−1^·K^−1^) [61], and Antimonene (5 W·m^−1^·K^−1^) [56]. 

### 3.6. Thermoelectric Figure of Merit

**Figure 9:**
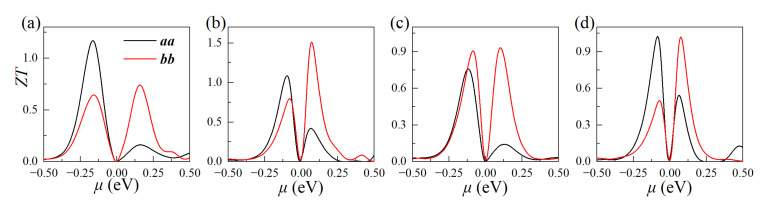
*ZT* values of the monolayer: (**a**) CuSbS_2_; (**b**) CuSbSe_2_; (**c**) CuBiS_2_; and (**d**) CuBiSe_2_ at 300 K.

Finally, we fitted the thermoelectric figure of merit (*ZT*) of these four monolayers at 300 K, as shown in Figure 9 (see Figures S8 and S9 for more details about the thermoelectric properties at without SOC functional). Obviously, the monolayers exhibit higher p-type *ZT* values in *a*-axis, while higher n-type *ZT* in *b*-axis, which is consistent with the results of higher hole mobility in the *a*-axis, while higher electron mobility in *b*-axis. As listed in Table 4, the monolayers simultaneously exhibit high *ZT* values for p-type of 0.91~1.17, and n-type of 0.74~1.53 at 300 K, which are higher than or comparable to those of many 2D thermoelectric materials, such as Pd_2_Se_3_ (0.9) [6], Tellurene (0.6) [62], InSe (0.5) [63], and SnSe (0.5) [64].

## 4. Conclusions

In this work, we investigated the stability, mechanical, electrical, and phonon transport properties of 2D CuMN_2_ (M = Sb, Bi; N = S, Se). We found that monolayers possess the acceptable cleavage energies of 0.68~0.93 J/m^2^, and narrow band-gaps of 0.57~1.10 eV, respectively. Based on the acoustic phonon-limited method, we revealed that the electron and hole mobility are basically in the range of 10^2^~10^3^ cm^2^·V^−1^·s^−1^. Besides, they also have high electrical conductivity of 10^6^~10^7^ Ω^−1^·m^−1^, high *PF* of 18.04~47.34 mW·K^−2^·m^−1^ at 300 K. Furthermore, due to the stronger phonon anharmonic interactions and low phonon lifetimes, their lattice thermal conductivities are as low as 0.23~3.30 Wm^−1^ K^−1^. As a result, all the monolayers simultaneously exhibit high *ZT* values for p-type of 0.91~1.17, and n-type of 0.74~1.53 at 300 K, indicating that they have potential applications in nano-electronic and thermoelectric devices.

## Figures and Tables

**Table 1:** The structural parameters, buckling height *h*, cleavage energies *E*_f_, and band gaps *E*_g_ of 2D CuMN_2_ (M = Sb, Bi; N = S, Se).

Materials	*a* (Å)	*b* (Å)	*h* (Å)	*E*_f_ (J/m^2^)	*E*_g_ (eV)		
					PBE	PBE + SOC	HSE 06 + SOC
CuSbS_2_	6.15	3.81	5.23	0.72	0.38	0.37	1.10
CuSbSe_2_	6.48	4.03	5.43	0.68	0.20	0.19	0.68
CuBiS_2_	6.21	3.95	5.24	0.93	0.35	0.26	0.83
CuBiSe_2_	6.55	4.16	5.44	0.85	0.20	0.16	0.57

**Table 2:** The Elastic constants $C_{ij}$, the maximums for Young’s modulus *Y* and Poisson’s ratio *υ*, and Debye temperature $\Theta_{D}$ of 2D CuMN_2_ (M = Sb, Bi; N = S, Se).

Materials	C_11_ (N/m)	C_12_ (N/m)	C_22_ (N/m)	C_66_ (N/m)	Y (N/m)	υ	$\Theta_{D}$ (K)
CuSbS_2_	66.53	−0.69	33.05	16.44	66.52	0.14	90.10
CuSbSe_2_	66.58	7.92	29.28	13.81	64.44	0.29	77.30
CuBiS_2_	58.26	−1.34	31.82	15.04	58.20	0.14	106.80
CuBiSe_2_	60.83	8.49	33.70	12.94	58.69	0.34	75.40

**Table 3:** The carrier effective mass (m*/*m*_0_), deformation potential constant (*E_l_*/eV), plane stiffness (*C*^2D^/N·m^−1^), hole (*μ_h_*/cm^2^·V^−1^·s^−1^) and electron mobility (*μ_e_*/cm^2^·V^−1^·s^−1^), and relaxation time (*τ*/fs) of the monolayers under PBE + SOC functional at 300 K.

Materials	Direction	Type	m*	*C* ^2D^	*E_l_*	DPT	APT	
						*μ*	*μ*	*τ*
CuSbS_2_	*a*-axis	electron	1.83	63.50	1.38	584.78	206.92	215.29
		hole	0.77		0.75	4562.47	1661.49	732.56
	*b*-axis	electron	0.24	34.15	3.27	424.59	895.32	122.89
		hole	0.60		3.05	192.61	684.04	232.33
CuSbSe_2_	*a*-axis	electron	1.97	51.83	2.08	176.33	166.61	186.53
		hole	0.93		1.22	1085.34	248.49	132.20
	*b*-axis	electron	0.27	26.30	2.28	532.97	937.12	146.58
		hole	0.57		4.48	66.84	196.34	63.82
CuBiS_2_	*a*-axis	electron	3.11	53.75	2.40	54.62	58.96	104.40
		hole	0.78		1.20	1511.80	820.27	366.20
	*b*-axis	electron	0.44	33.55	1.83	415.29	412.27	103.07
		hole	0.57		2.08	430.36	745.06	242.74
CuBiSe_2_	*a*-axis	electron	0.99	44.13	2.67	138.35	196.09	110.83
		hole	2.83		0.89	495.73	176.14	283.82
	*b*-axis	electron	0.93	27.99	1.94	178.15	213.08	112.36
		hole	0.25		2.63	404.18	1071.33	153.80

**Table 4:** The maximums of Seebeck coefficient *S* (mV·K), electron thermal conductivity $\kappa_{e}$ (W·m^−1^·K^−1^), electrical conductivity *σ* (×10^6^ Ω^−1^·m^−1^), lattice thermal conductivity $\kappa_{l}$ (W·m^−1^·K^−1^), *PF* (mW·K^−2^·m^−1^) and *ZT* values in the chemical potential of −1 eV~1 eV at 300 K.

Monolayers	Direction	Carriertype	*S*	*σ*	$\kappa_{e}$	$\kappa_{l}$	*PF*	*ZT*
CuSbS_2_	*aa*	p-type	0.46	45.96	329.64	2.96	47.34	1.17
		n-type	0.33	4.66	31.01		5.07	0.16
	*bb*	p-type	0.36	26.56	191.40	2.38	15.50	0.65
		n-type	0.43	20.47	147.55		24.43	0.74
CuSbSe_2_	*aa*	p-type	0.24	8.52	60.52	0.49	8.05	1.09
		n-type	0.15	3.80	24.82		10.84	0.91
	*bb*	p-type	0.17	7.49	53.62	0.12	3.89	0.84
		n-type	0.23	21.64	145.33		20.42	1.53
CuBiS_2_	*aa*	p-type	0.31	17.87	125.18	3.30	19.79	0.76
		n-type	0.23	1.84	12.27		1.96	0.14
	*bb*	p-type	0.24	28.63	204.23	1.32	17.45	0.91
		n-type	0.29	7.96	57.16		11.15	0.94
CuBiSe_2_	*aa*	p-type	0.19	15.79	108.79	0.23	17.93	1.03
		n-type	0.14	2.40	14.66		5.00	0.55
	*bb*	p-type	0.13	18.19	128.59	0.21	7.74	0.50
		n-type	0.18	8.33	57.81		18.04	1.03

## Data Availability

The data presented in this study are available on request from the corresponding authors. The data are not publicly available due to ongoing research in the project.

## Supplementary Materials

The following supporting information can be downloaded at: https://www.mdpi.com/article/10.3390/ma15196700/s1, Table S1: The cleavage energies, Figure S1: the Electron Localization Functions, Figure S2: the ab initio molecules dynamics simulation, Table S2: Bader charge analysis results, Figure S3: the band structures at PBE functional without and with SOC functional, Figure S4: the partial density of states, Figure S5, Table S3, Figure S6: the details on carrier mobility calculations, Figure S7: the MLWFs calculated by Wannnier90 code, Figures S8 and S9: the thermoelectric properties of monolayer CuMN_2_ (M = Sb, Bi; N = S, Se) without SOC functional.
