# Supplementary material for: Excellent Thermoelectric Performance of 2D CuMN2 (M = Sb, Bi; N = S, Se) at Room Temperature
Source: Materials (Basel). 2022 Sep 27;15(19):6700. doi: 10.3390/ma15196700 (PMC9572028; doi:10.3390/ma15196700)
Supplement: Supplementary file 1 [file materials-15-06700-s001.zip › materials-1911015-supplementary.pdf]

Supplementary Materials for

Excellent thermoelectric performance of 2D $CuMN_2$ (M = Sb, Bi; N = S, Se) at room temperature

Wenyu Fang[1, 2†], Yue Chen[1†], Kuan Kuang[1], Mingkai Li[1*]

[1] Ministry-of-Education Key Laboratory of Green Preparation and Application for Functional Materials, Hubei Key Lab of Ferro & Piezoelectric Materials and Devices, Hubei Key Laboratory of Polymer Materials, and School of Materials Science & Engineering, Hubei University, Wuhan 430062, China

[2] Public Health and Management School, Hubei University of Medicine, Shiyan 442000, China

[†]These authors contributed equally to this work.

*Corresponding authors.

E-mail addresses: mkli@hubu.edu.cn (M. K. Li).

Calculation details

We carried out our calculations in Vienna Ab initio Simulation Package (VASP), in which the Generalized Gradient Approximation (GGA) and Perdew - Burke - Ernzerhof (PBE) functional was adopted to exchange-correlation approximation. The kinetic energy cut-off for plane-

wave basis was set as 500 eV, the convergence criteria for total energy and ion were set as 1.0 × 7 eV and -0.005 eV, respectively. The k-mesh of 20 × 20 × 1 was adopted in self-consistent calculation, and the k-points path in first Brillouin region for band structure and phonon dispersion was set as Γ - X - S - Y - Γ, as shown in Figure 1(e). Also, we selected 4 × 5 × 1 supercell (160 atoms) to calculate the phonon dispersion and lattice thermal conductivity with 4 × 4 × 1 q- points mesh.

Carrier mobility

The carrier mobility was first calculated by the method of deformation potential theory (DPT) proposed by Bardeen and Shockley, which can be expressed as:

$$\mu_{2D} = \frac{e\hbar^3 C^{2D}}{k_B T m^* m_l^* (E_l^i)^2} \quad \text{(S1)}$$

Where μ_{2D}, $\hbar$, k_B, and T are in-plane mobility, the reduced Planck constant, Boltzmann constant, and temperature. m^* can be obtained by using the second derivative of the energy near the VBM and CBM with respect to the wave vector, i.e, $m^* = \hbar^2/(\frac{\partial^2 E}{\partial k^2})$, here E and k are the energy and wave vector, respectively, and m_l^* is the geometric average of the effective mass along the *a* and *b* directions, i.e, $m_l^* = \sqrt{m_a^* m_b^*}$.

And then, we adopted the acoustic phonon-limited method (APM) proposed by Lang *et al.* in 2016, which can be applied to anisotropic 2D materials, and its calculation methods are as follows:

$$\mu_x = \frac{e\hbar^3}{k_B T (m_x)^{\frac{3}{2}} (m_y)^{\frac{1}{2}}} \left(\frac{A+B-\sqrt{A^2-B^2}}{B\sqrt{A^2-B^2}} \right) \times \left(\frac{I+J-\sqrt{I^2-J^2}}{J\sqrt{I^2-J^2}} \right) \quad \text{(S2)}$$

$$A = \bar{E}_l^2 + \frac{(\Delta E_l)^2}{2}, \; B = \bar{E}_l \Delta E_l \quad \text{(S3)}$$

$$\bar{E}_l = \frac{E_{lx}+E_{ly}}{2}, \; \Delta E_l = \frac{E_{ly}-E_{lx}}{2} \quad \text{(S4)}$$

$$\bar{C} = \frac{C_{11}+C_{22}}{2}, \; \Delta C = \frac{C_{22}-C_{11}}{2} \quad \text{(S5)}$$

$$I = \frac{1}{\sqrt{\bar{C}^2-(\Delta C)^2}}, \; J = \frac{\bar{C}}{\Delta C} \left(\frac{1}{\bar{C}} - \frac{1}{\sqrt{\bar{C}^2-(\Delta C)^2}} \right) \quad \text{(S6)}$$

Table S1 The total energies, and cleavage energies (E_f) for the monolayers.

Monolayers	Bulk (eV)	Mono (eV)	S (Å^2)	a (Å^2)	b (Å^2)	E_f (J/m^2)
$CuSbS_2$	-289.32	-143.64	22.74	6.04	3.77	0.72
$CuSbSe_2$	-371.53	-184.69	25.20	6.34	3.97	0.68
$CuBiS_2$	-453.71	-225.46	23.98	6.18	3.88	0.93
$CuBiSe_2$	-536.26	-266.74	26.31	6.47	4.07	0.85

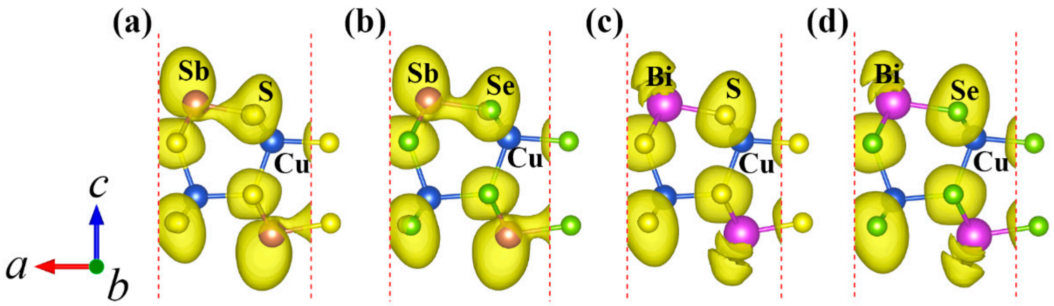

Figure S1 The Electron Localization Functions (ELFs) for monolayer (a) $CuSbS_2$, (b) $CuSbSe_2$, (c) $CuBiS_2$, and (d) $CuBiSe_2$.

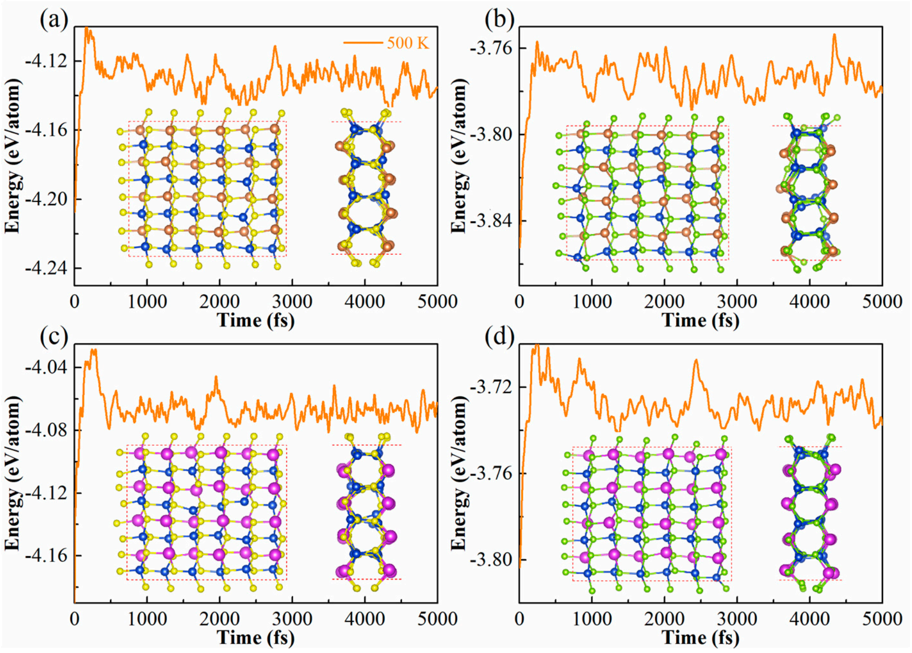

Figure S2 The energy variations as well as the top/side view of the snapshots from the *ab initio* molecules dynamics simulation for the monolayer (a) $CuSbS_2$, (b) $CuSbSe_2$, (c) $CuBiS_2$, and (d) $CuBiSe_2$ at 500 K.

Table S2 Bader charge analysis results for the monolayers.

Monolayers	Cu (e)	Sb/Bi (e)	S/Se (e)
$CuSbS_2$	0.49	0.99	0.74
$CuSbSe_2$	0.37	0.76	0.57
$CuBiS_2$	0.48	0.98	0.73
$CuBiSe_2$	0.36	0.79	0.56

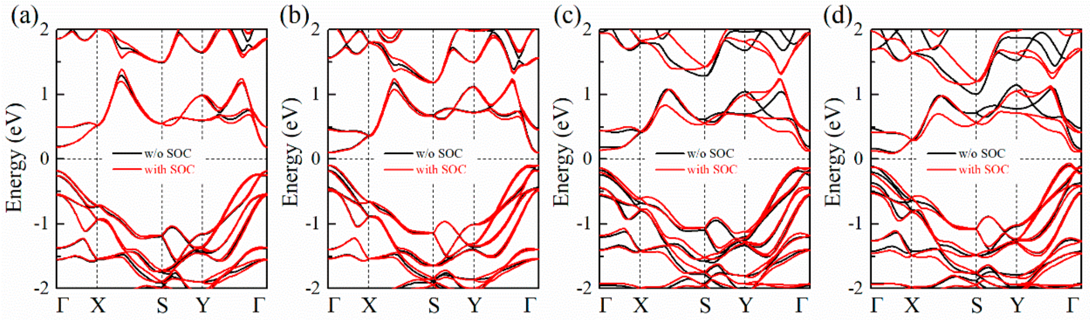

Figure S3 The band structures at PBE functional without (black line) and with SOC (red line) for monolayer (a) $CuSbS_2$, (b) $CuSbSe_2$, (c) $CuBiS_2$, and (d) $CuBiSe_2$.

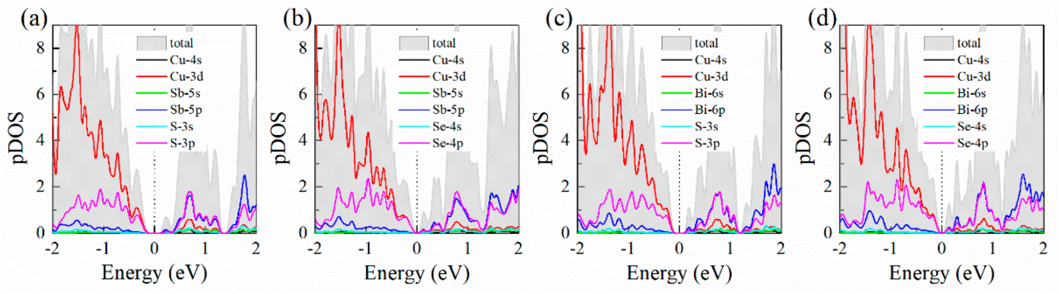

Figure S4 The partial density of states of monolayer (a) $CuSbS_2$, (b) $CuSbSe_2$, (c) $CuBiS_2$, and (d) $CuBiSe_2$.

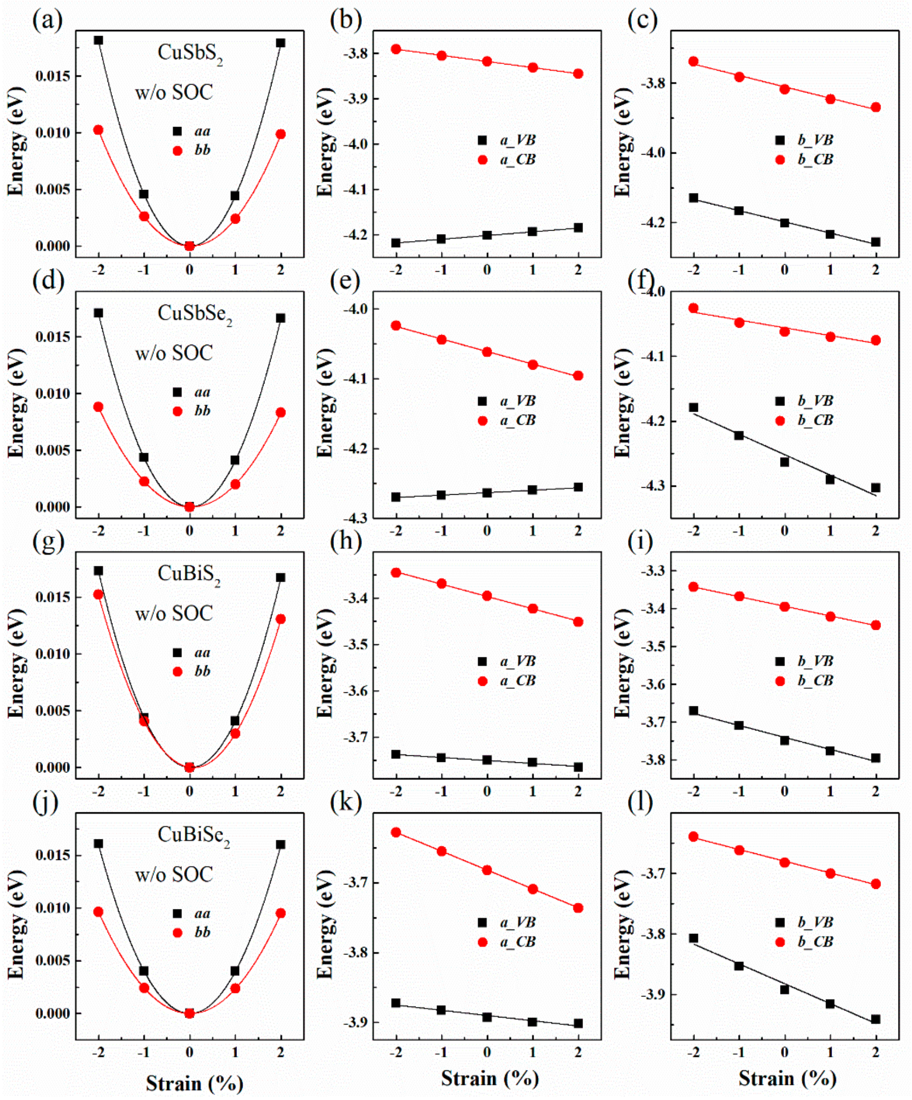

Figure S5 (a, d, g, j) Total energy difference between the unstrained and strained $CuMN_2$ (M = Sb, Bi; N = S, Se) monolayer along the *a* and *b* directions; (b, e, h, k) Energy shift of VBM and CBM for the monolayers with respect to the lattice dilation and compression along *a* direction; (c, f, i, l) Energy shift of VBM and CBM for the monolayers with respect to the lattice dilation and compression along the *b* direction. These results are calculated by GGA+PBE functional without SOC.

Table S3 The effective mass (m^*/m_0), elastic modulus (C^{2D}/N·m^{-1}), deformation potential constant (E_l/eV), electron (μ_e /cm^2·V^{-1}·s^{-1}) and hole mobility (μ_h /cm^2·V^{-1}·s^{-1}), and relaxation time (τ /fs) of monolayers at PBE functional without SOC.

Monolayers	Direction	Type	m^*	C^{2D}	E_l	DPT	APM	
						μ	μ	τ
$CuSbS_2$	*a*-axis	electron	2.12	61.64	1.35	477.09	169.72	204.64
		hole	0.83		0.82	3175.85	566.57	268.12
	b-axis	electron	0.24	34.38	3.26	403.56	850.67	115.99
		hole	0.66		3.22	145.41	349.35	130.61
$CuSbSe_2$	*a*-axis	electron	1.68	51.83	1.80	310.01	407.39	389.20
		hole	0.69		0.35	19458.67	1452.69	567.04
	b-axis	electron	0.26	26.31	1.20	2329.38	2688.94	390.42
		hole	0.66		3.16	125.11	497.50	188.05
$CuBiS_2$	a-axis	electron	2.01	55.59	2.66	101.42	90.99	103.93
		hole	0.93		0.64	5209.96	638.95	338.54
	b-axis	electron	0.34	46.17	2.55	546.82	478.59	91.63
		hole	0.38		3.17	430.71	736.19	159.72
$CuBiSe_2$	*a*-axis	electron	0.76	47.03	2.70	335.64	449.27	194.25
		hole	0.77		0.75	4119.16	764.24	336.58
	b-axis	electron	0.38	28.09	1.94	772.63	911.09	198.00
		hole	0.40		3.28	247.06	713.95	163.73

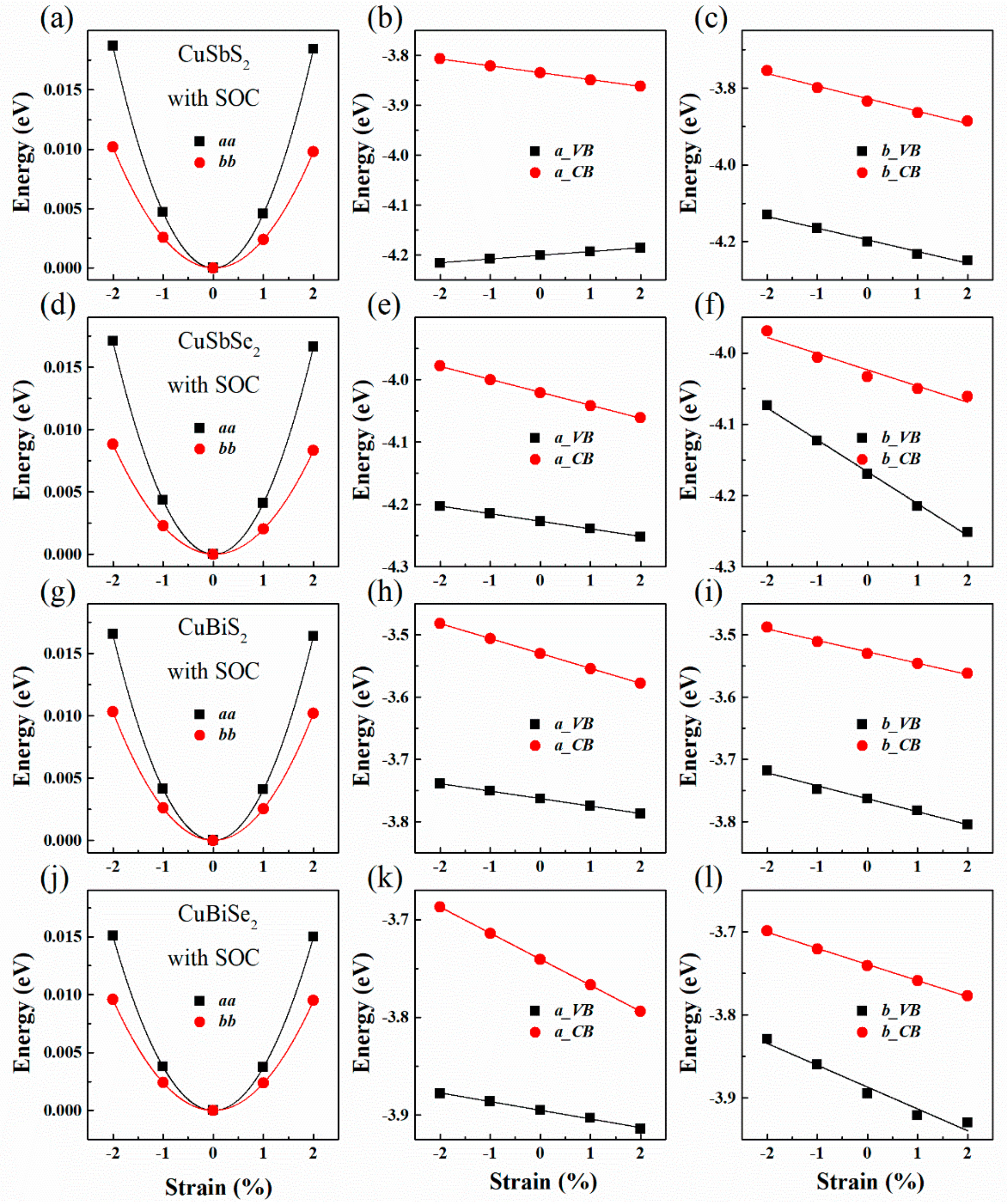

Figure S6 (a, d, g, j) Total energy difference between the unstrained and strained $CuMN_2$ (M = Sb, Bi; N = S, Se) monolayer along the *a* and *b* directions; (b, e, h, k) Energy shift of VBM and CBM for the monolayers with respect to the lattice dilation and compression along *a* direction; (c, f, i, l) Energy shift of VBM and CBM for the monolayers with respect to the lattice dilation and compression along the *b* direction. These results are calculated by GGA+PBE functional with SOC.

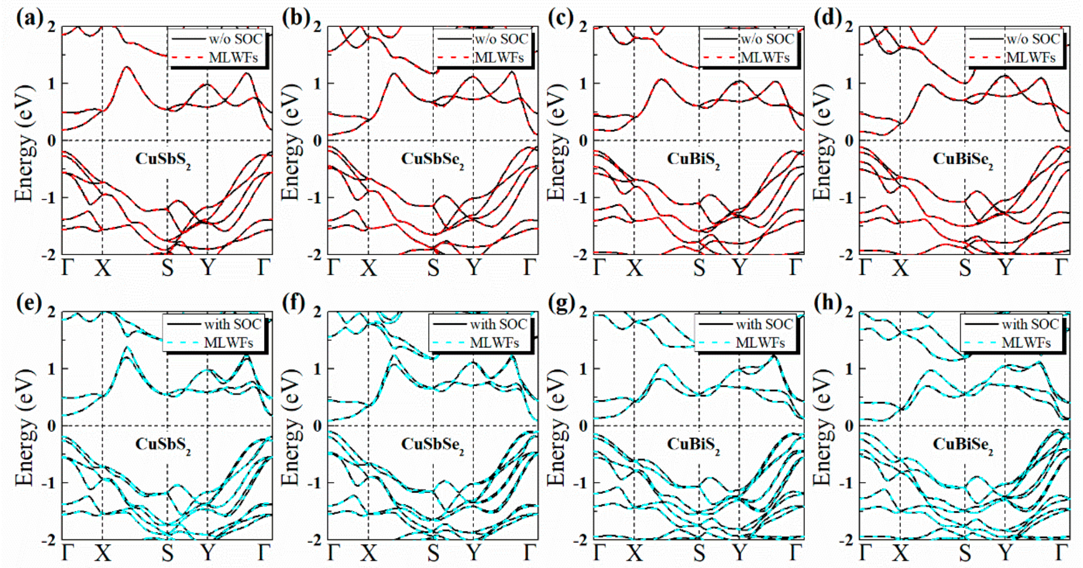

Figure S7 The band structures at GGA+PBE functional and MLWFs calculated by Wannier90 code. The close coincidence of two curves suggests that the Boltzmann electron transport equation solved by wannier90 code is accurate and reliable.

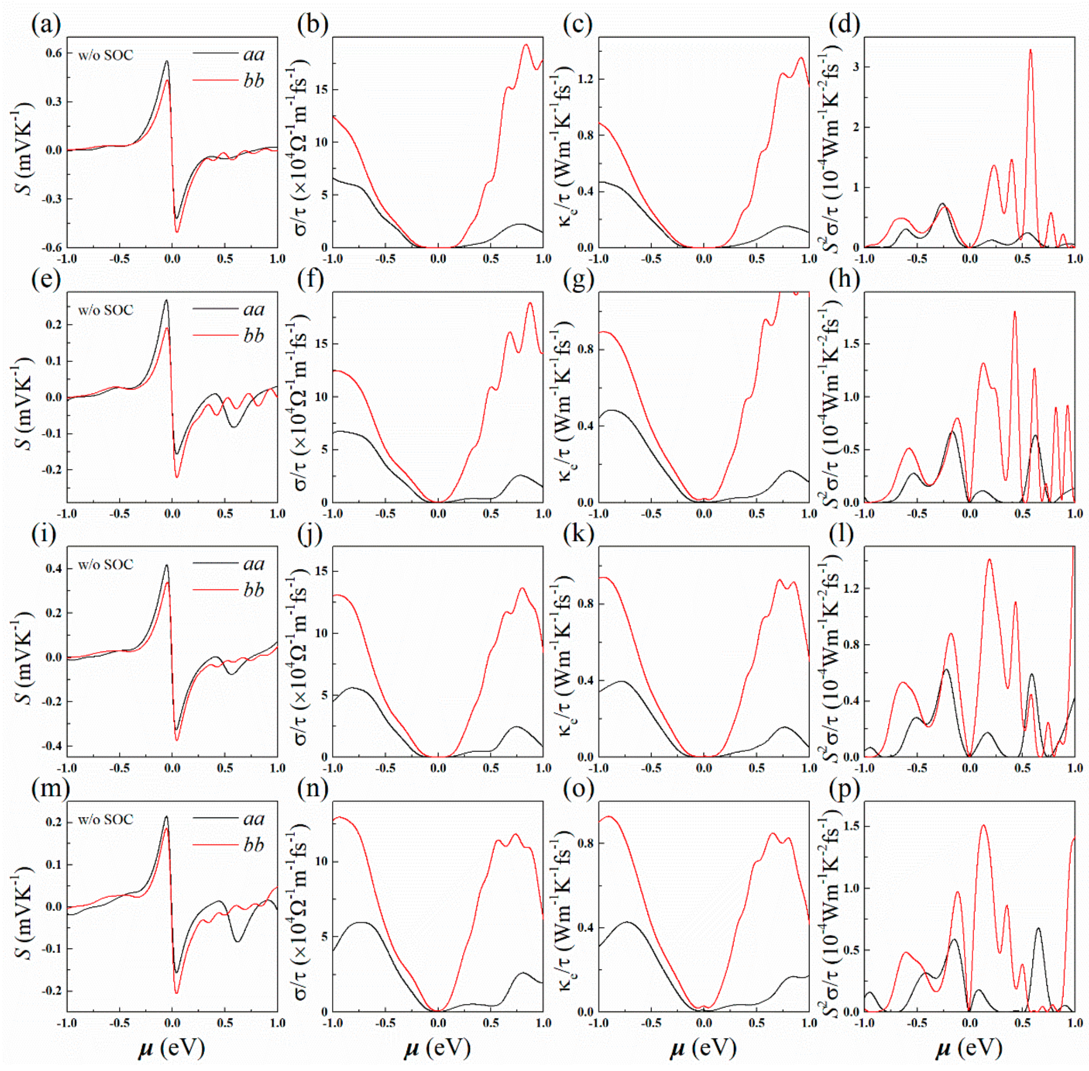

Figure S8 Electron transport properties of 2D $CuMN_2$ (M = Sb, Bi; N = S, Se). (a, e, i, m) seebeck coefficients, (b, f, j, n) electrical conductivities, (c, g, k, o) electron thermal conductivities and (d, h, i, p) Power factors along *a*- (black line) and *b*- directions (red line) without SOC functional.

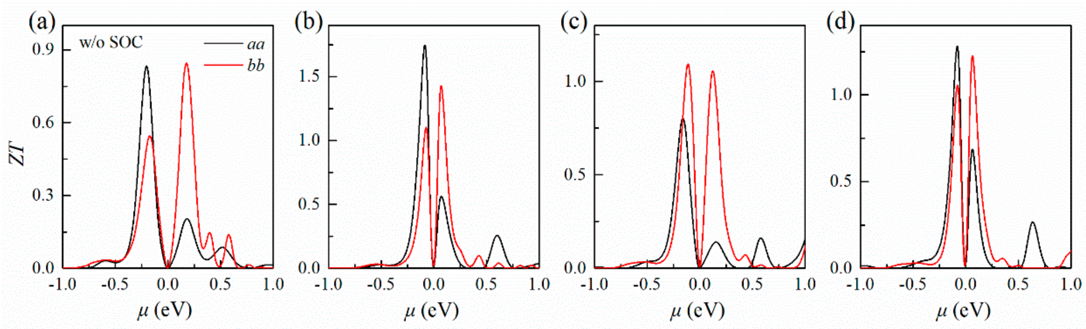

Figure S9 *ZT* values of the monolayer (a) $CuSbS_2$, (b) $CuSbSe_2$, (c) $CuBiS_2$, and (d) $CuBiSe_2$ without SOC functional.
